# Unusual left‐sided variant orientation of the slow conduction zone in adenosine‐sensitive atrial tachycardia

**DOI:** 10.1002/joa3.70015

**Published:** 2025-02-07

**Authors:** Shuhei Arai, Taku Asano, Yuki Takai, Yuya Nakamura, Toshiro Shinke

**Affiliations:** ^1^ Division of Cardiology, Department of Medicine Showa University School of Medicine Shinagawa‐ku Japan

**Keywords:** adenosine sensitive, atrial tachycardia, catheter ablation, entrainment, left atrial appendage

## Abstract

Adenosine‐sensitive atrial tachycardia (AT) is typically associated with a slow conduction zone (SCZ) near the atrioventricular node or tricuspid annulus. We report an unusual case of adenosine‐sensitive AT with a SCZ located between the left atrial appendage and the left atrial anterior septum, which was successfully ablated 9.2 mm from the left atrial earliest activation site. This case highlights the importance of advanced mapping and entrainment techniques in the identification and management of rare left‐sided SCZ variants of adenosine‐sensitive AT.

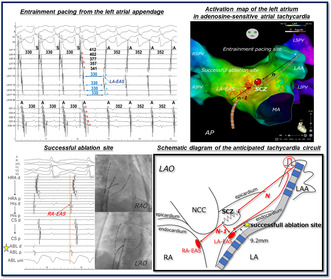

Adenosine‐sensitive atrial tachycardia (AT) is thought to result from a micro‐re‐entry mechanism. Typically, adenosine‐sensitive AT arises near the atrioventricular node (AVN) and tricuspid annulus and can be effectively and safely ablated at the entrance to the slow conduction zone (SCZ) using a manifest entrainment‐guided approach, while avoiding atrioventricular block.^1^ While adenosine‐sensitive AT predominantly originates from these areas, rare cases have been reported involving the left atrium (LA).^2^ We present the case of adenosine‐sensitive atrial tachycardia with a SCZ between the left atrial appendage (LAA) and anterior septum of the LA.

A 51‐year‐old woman without any overt structural heart disease was referred to our hospital for catheter ablation of a symptomatic long RP narrow QRS tachycardia. Multi‐electrode catheters were placed in the high right atrium (HRA), His bundle region, coronary sinus (CS), and right ventricular apex (RVA). During sinus rhythm (Figure 1A), atrio‐His interval and His‐ventricular interval were 110 and 48 ms, respectively. No ventriculo‐atrial (VA) conduction was observed. During an isoproterenol infusion, atrial extrastimuli consistently induced the clinical tachycardia with a TCL of 358 ms without an atrio‐His interval jump (Figure 1B). The 12‐lead ECG during tachycardia demonstrated P‐wave morphology characterized by negative or isoelectric deflections in leads I and aVL, and positive deflections in leads II, III, aVF, and V1 (Figure 1A). The right atrium (RA) activation mapping was performed during tachycardia using a high‐density multipolar electrode mapping catheter (OCTARAY; Biosense Webster, Diamond Bar, CA, USA). The earliest activation site (EAS) was the right atrial postero septum, 25 mm posterior to the His bundle potential recording site (Figure 1C). VA dissociation was observed during overdrive pacing from the RVA, excluding orthodromic reciprocating tachycardia with any accessory pathways. The VA interval after differential atrial overdrive pacing from the CS proximal, CS distal, and HRA showed no VA linking,^3^ suggesting AT (Figure S1). Furthermore, when the atrial extrastimuli interval was shortened, the His bundle potential was reset for the first time, and the pre‐ and post‐AA intervals were unchanged, resulting in the diagnosis of AT (Figure 2A).^3^ During tachycardia, rapid intravenous administration of 1 mg of adenosine triphosphate prolonged the TCL and terminated tachycardia reproducibly without affecting atrioventricular conduction (Figure 2B). Therefore, the patient was diagnosed with adenosine‐sensitive AT. Overdrive pacing was performed from multiple locations in the RA during tachycardia, RA‐EAS was captured antidromically following pacing from the right atrial appendage, low lateral, and high posteroseptal regions of the RA and CS. Thus, a transseptal puncture was performed. The LA activation map showed a centrifugal pattern and the EAS was located at the anterior septum of the LA (Video S1). The left atrial earliest activation site (LA‐EAS) preceded the RA‐EAS by 10 ms. For the simultaneous recording of the LAA and LA‐EAS local electrograms, a linear multipolar electrode catheter was placed (Figure S2). The LA‐EAS was captured orthodromically following overdrive pacing from the LAA (Figure 3A,B; Figure S3). This indicated that an entry of the SCZ was located between the LAA and LA‐EAS. Ablation was started at a site approximately 15 mm from the LA‐EAS along a hypothetical straight line connecting the LAA to the LA‐EAS. If tachycardia termination was not achieved, the ablation was progressively advanced closer to the LA‐EAS. As a result, a radiofrequency application at 9.2 mm away from the LA‐EAS toward the LAA successfully terminated the tachycardia within 6 s (Figure S4). This application was performed at 35 W with a contact force of 10–15 g, and energy delivery was continued for a total of 60 s, which rendered any further tachycardia noninducible. During the 6‐month follow‐up period, there was no recurrence of tachycardia, and the patient's palpitations had completely resolved.

**FIGURE 1:**
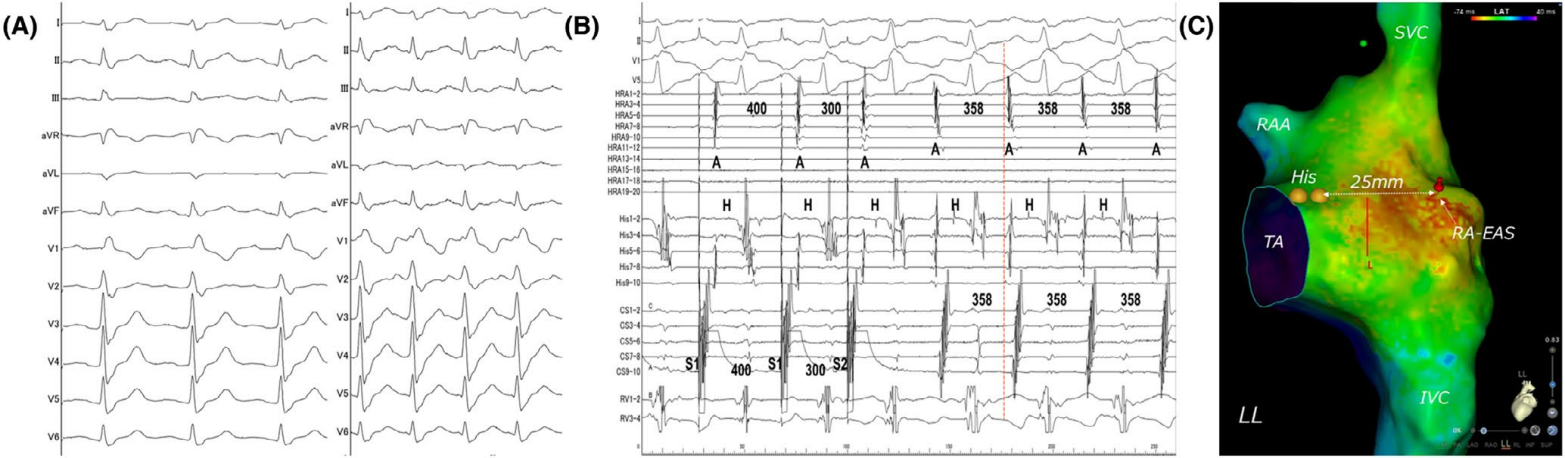
(A) The 12‐lead ECG displays sinus rhythm on the left and tachycardia on the right. Compared to sinus rhythm, the P‐wave duration during tachycardia is narrower, suggesting that its origin is in close proximity to the atrial septum. During tachycardia, demonstrated P‐wave morphology characterized by negative or isoelectric deflections in leads I and aVL, and positive deflections in leads II, III, aVF, and V1. (B) Programmed atrial extrastimuli with an S1–S2 coupling interval of 300 ms induced clinical tachycardia with a cycle length of 358 ms without an atrio‐His jump. The earliest atrial activation was recorded almost simultaneously by HRA and His bundle electrodes (indicated by the orange dotted line). (C) 3D mapping image of atrial tachycardia. The activation map of the right atrium revealed a centrifugal pattern, with the earliest activation located 25 mm posterior to the His bundle. CS, coronary sinus; His, his bundle; HRA, high right atrium; IVC, inferior vena cava; LL, left lateral view; RA‐EAS, right atrial earliest activation site; RAA, right atrial appendage; RV, right ventricle; SVC, superior vena cava; TA, tricuspid annulus.

**FIGURE 2:**
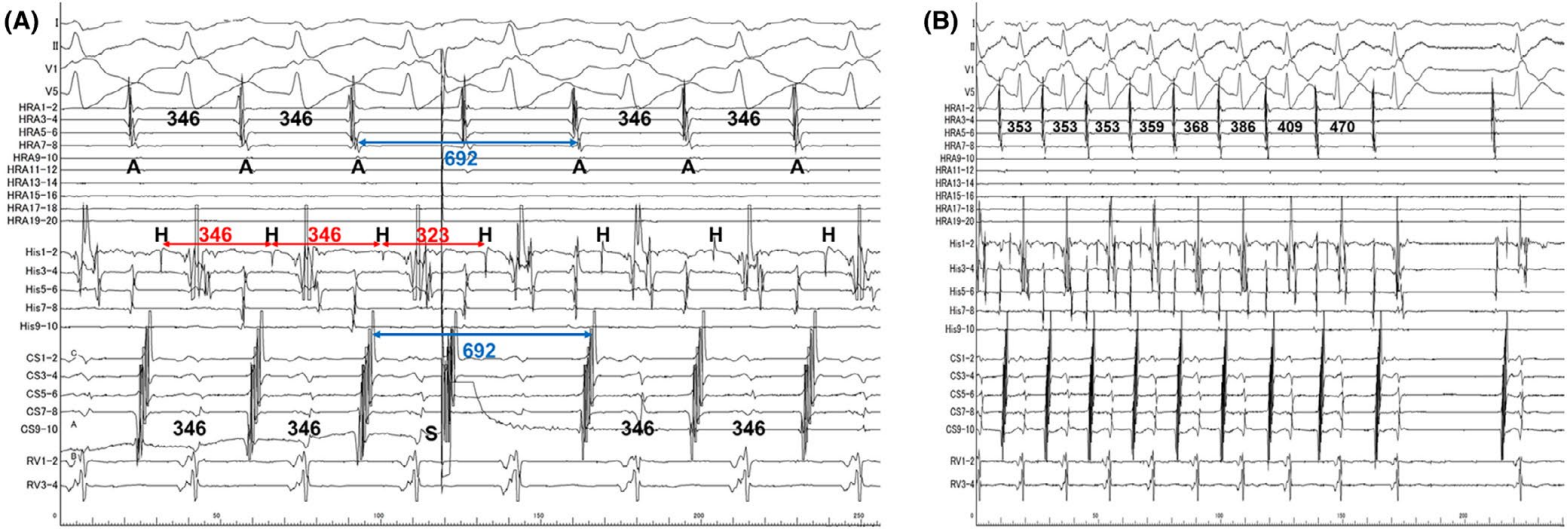
(A) The values shown on the intracardiac ECG represent atrial cycle lengths or His‐His intervals. As the atrial extrastimuli interval was shortened, the His bundle potential was reset for the first time, with no change observed in the pre‐ and post‐AA intervals. (B) Rapid intravenous administration of 1 mg of adenosine triphosphate prolonged the tachycardia cycle length and terminated tachycardia without affecting atrioventricular conduction. CS, coronary sinus; Hi, his bundle; HRA, high right atrium; RV, right ventricle.

**FIGURE 3:**
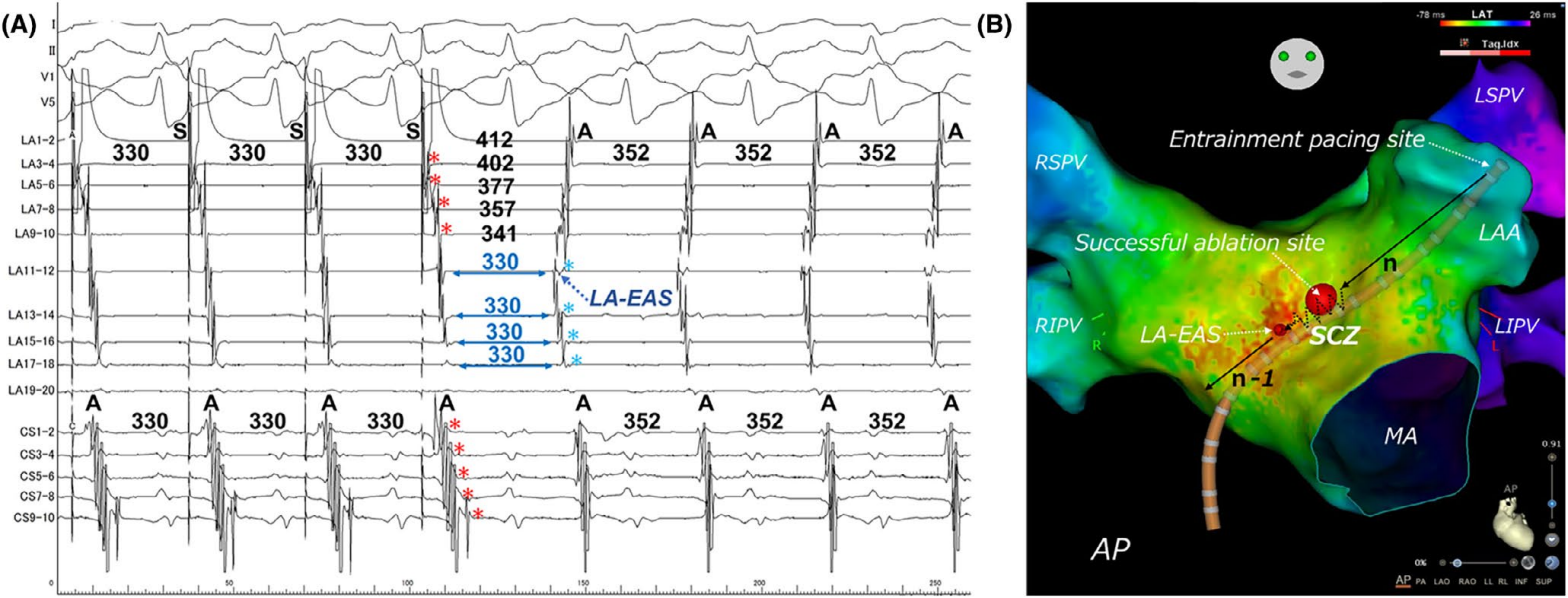
(A) Entrainment pacing from the LAA (LA1‐2). The LA‐EAS was located at the LA11‐12 electrodes. At this time, the CS catheter had been advanced deeper compared to the baseline, leading to an atrial activation sequence of CS distal‐to‐proximal. The cycle lengths of the tachycardia and pacing were 352 and 330 ms, respectively. The blue asterisks represent the last orthodromic captured electrograms, while the red asterisks represent the last antidromic captured electrograms. (B) The activation map of the LA revealed that the earliest activation site was at anterior septum. The antidromic wavefront is shown at the *n*‐th pacing and the orthodromic wavefront is shown as (*n* − 1). The (*n* − 1) exits from the SCZ and captures the LA‐EAS, where the SCZ is located between the LAA and the LA‐EAS. The successful ablation site was 9.2 mm away from the LA‐EAS. AP, anteroposterior view; CS, coronary sinus; LA, left atrium; LAA, left atrial appendage; LA‐EAS, left atrial earliest activation site; LIPV, left inferior pulmonary vein; LSPV, left superior pulmonary vein; MA, mitral annulus; RIPV, right inferior pulmonary vein; RSPV, right superior pulmonary vein; SCZ, slow conduction zone.

The response to adenosine indicates that the SCZ of the AT circuit involves calcium channel‐dependent tissues,^1^ such as the retroaortic node and atrioventricular (AV) ring, which are similar to the AVN. The retroaortic node, likely from embryonic AV ring remnants, is a specialized atrial tissue and is considered a potential substrate for adenosine‐sensitive AT near the AVN,^4^ located in the interatrial septum posterior to the non‐coronary cusp (NCC). Yamabe et al. recently demonstrated manifest entrainment and orthodromic capture from the high posteroseptal regions of the RA to the EAS. This suggests that the SCZ is oriented towards the NCC and supports the potential for successful ablation from the NCC.^5^ In our patient, the RA‐EAS was antidromically captured from the high posteroseptal RA, suggesting a potentially reduced efficacy of treatments targeting the NCC. Therefore, we prioritized mapping the LA over the NCC. In contrast, the orthodromic capture of the LA‐EAS from the LAA revealed that the SCZ is located between the LA‐EAS and the LAA. As shown in Figure 3A, the region from the LAA to just before the LA‐EAS is captured antidromically, suggesting the absence of a SCZ on the endocardial side in this area. However, the orthodromic capture of the LA‐EAS indicates that the SCZ is likely located on the epicardial side, specifically on the left‐sided region of the interatrial septum (Figure 4). Based on this observation, it could be inferred that the tachycardia may originate from the left side of the retroaortic node anatomically. In other words, we do not believe that the origin of the tachycardia was the LA itself, but rather a micro re‐entry involving the myocardium within the interatrial septum between the NCC and the LA. If the activation map shows even a slight early activation in the LA compared to the RA, it may be useful to perform entrainment from various locations within the LA. If ablation in the left atrium had been ineffective, mapping of the NCC should have been considered. In the treatment of adenosine‐sensitive AT, it is crucial to consider the results of entrainment and 3D mapping to determine the most suitable site (RA, NCC or LA) from which to ablate.

**FIGURE 4:**
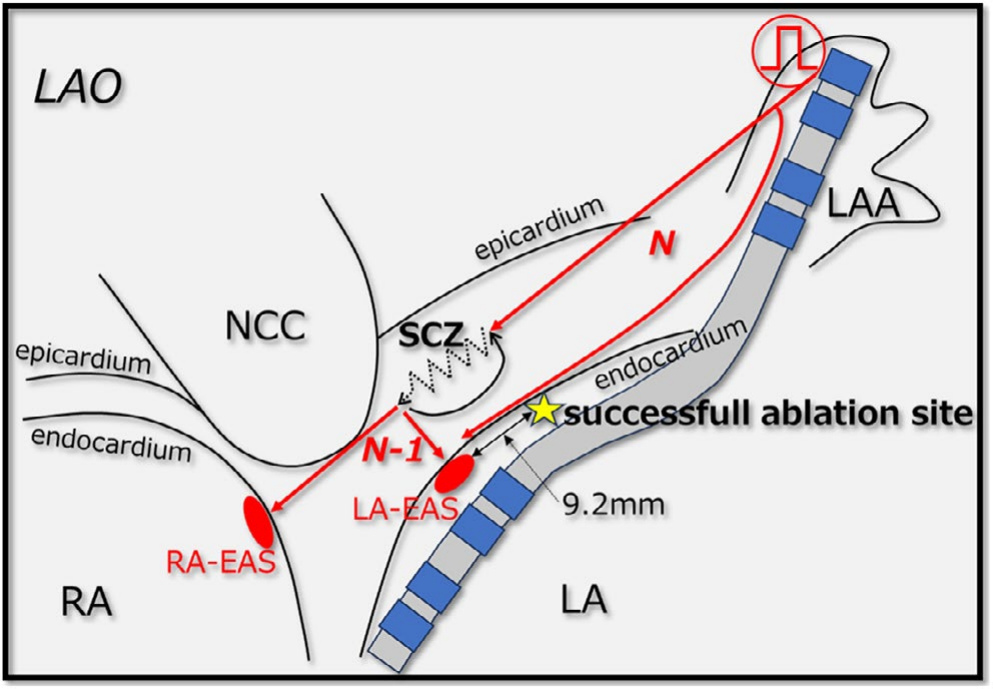
Schematic diagram of the anticipated tachycardia circuit and entrainment findings. LA, left atrium; LAA, left atrial appendage; LAO, left anterior oblique view; LA‐EAS, left atrial earliest activation site; NCC, non‐coronary cusp; RA, right atrium; RA‐EAS, right atrial earliest activation site; SCZ, slow conduction zone.

We experienced an unusual left‐sided variant orientation of the SCZ in adenosine‐sensitive AT. Manifest entrainment plays a critical role in diagnosing and identifying the orientation of the SCZ.

## FUNDING INFORMATION

This research did not receive any specific grant from funding agencies in the public, commercial, or not‐for‐profit sectors.

## CONFLICT OF INTEREST STATEMENT

Authors declare no conflict of interests for this article.

## PATIENT CONSENT STATEMENT

The patient has provided consent for publication.

## Supporting information


Figures S1–S4.

Video S1.
