# Supplementary material for: Unusual left‐sided variant orientation of the slow conduction zone in adenosine‐sensitive atrial tachycardia
Source: J Arrhythm. 2025 Feb 7;41(1):e70015. doi: 10.1002/joa3.70015 (PMC11803878; doi:10.1002/joa3.70015)
Supplement: Supplementary file 1 — Figures S1–S4. [file JOA3-41-e70015-s002.docx]

**Supplemental Figure**

(A)


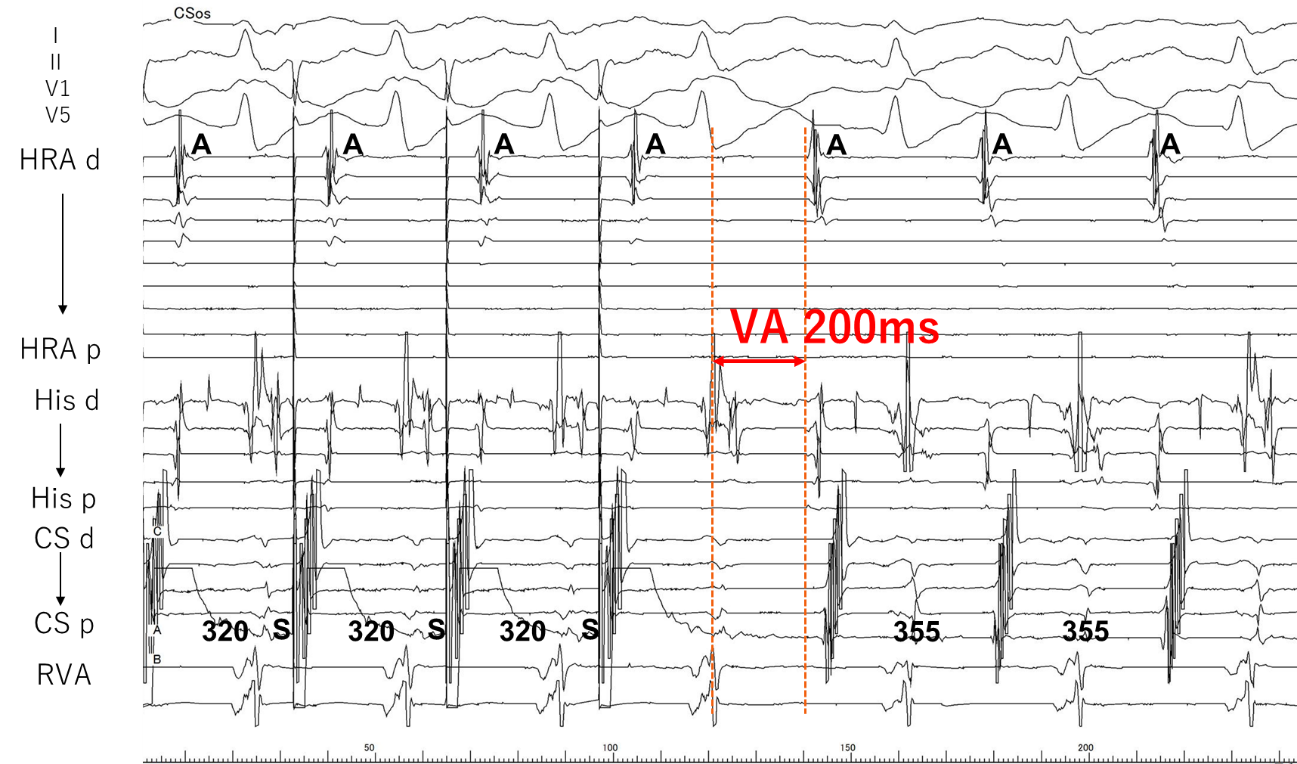


(B)


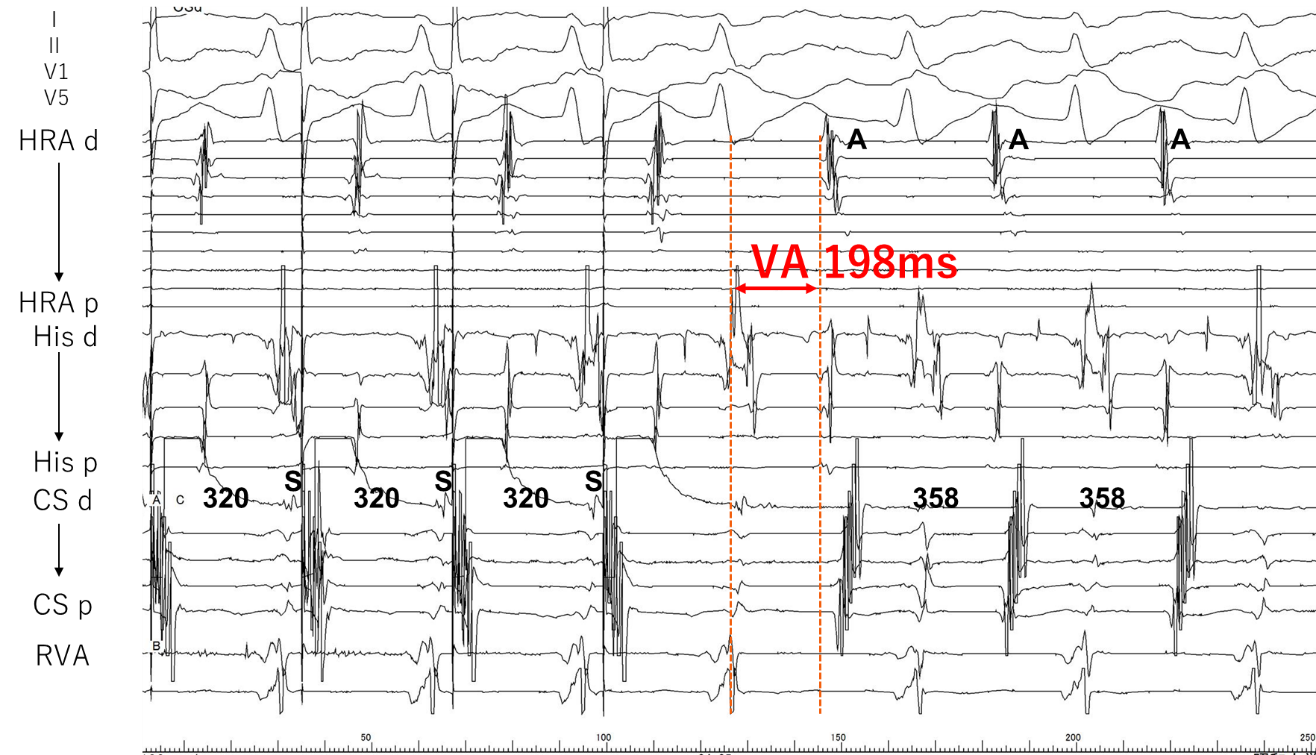


(C)


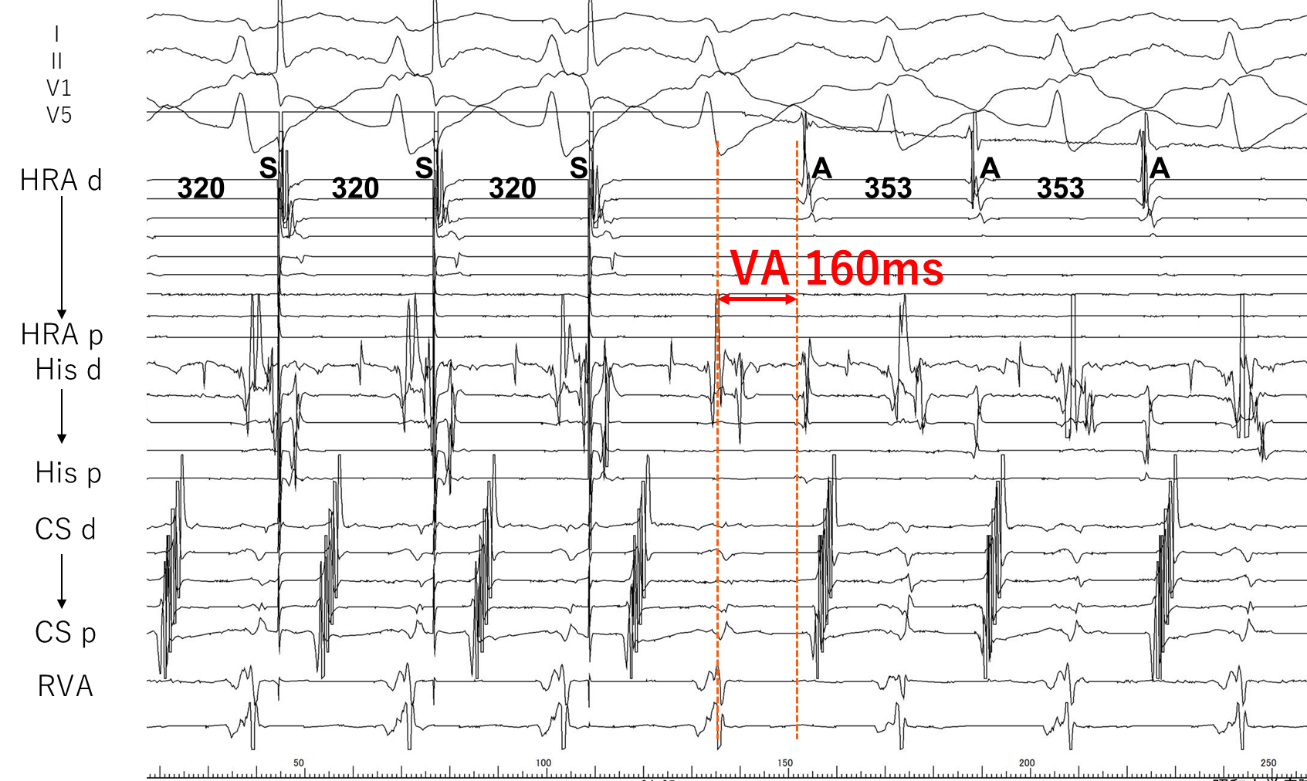


Supplemental Figure 1: Overdrive pacing during tachycardia from three sites (CS ostium, CS distal, and HRA) is shown. The pacing cycle length was 320 ms, and the post-pacing VA intervals were 200 ms, 198 ms, and 160 ms, respectively. The ΔVA interval was 40 ms (>20 ms), and no VA linking was observed. HRA = high right atrium; His = his bundle; CS = coronary sinus; RVA = right ventricular apex; d = distal; p = proximal.


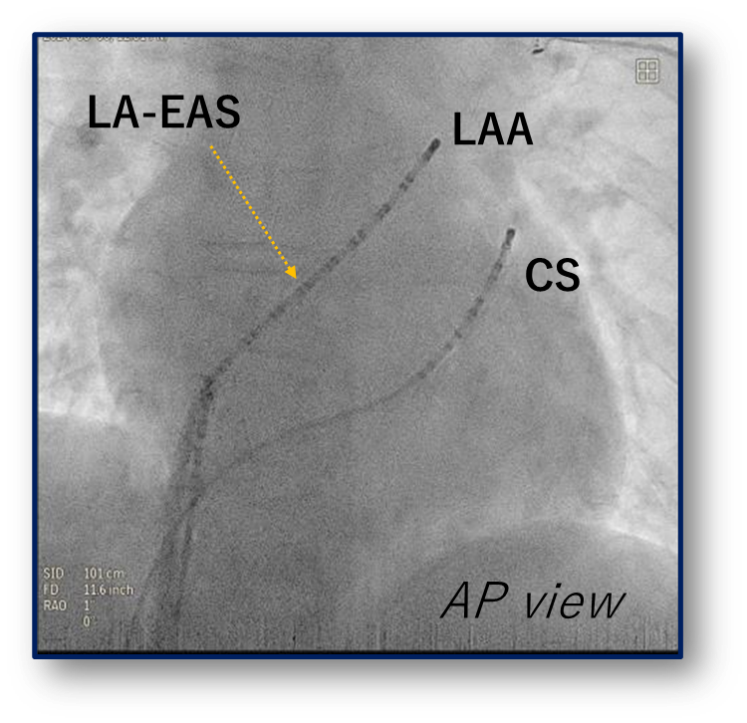


Supplemental Figure 2: Fluoroscopic image of the electrode catheter positioned to simultaneously record local electrograms from the LAA to the LA-EAS. CS = coronary sinus; LA-EAS = left atrial earliest activation site; LAA = left atrial appendage; AP = anteroposterior view.


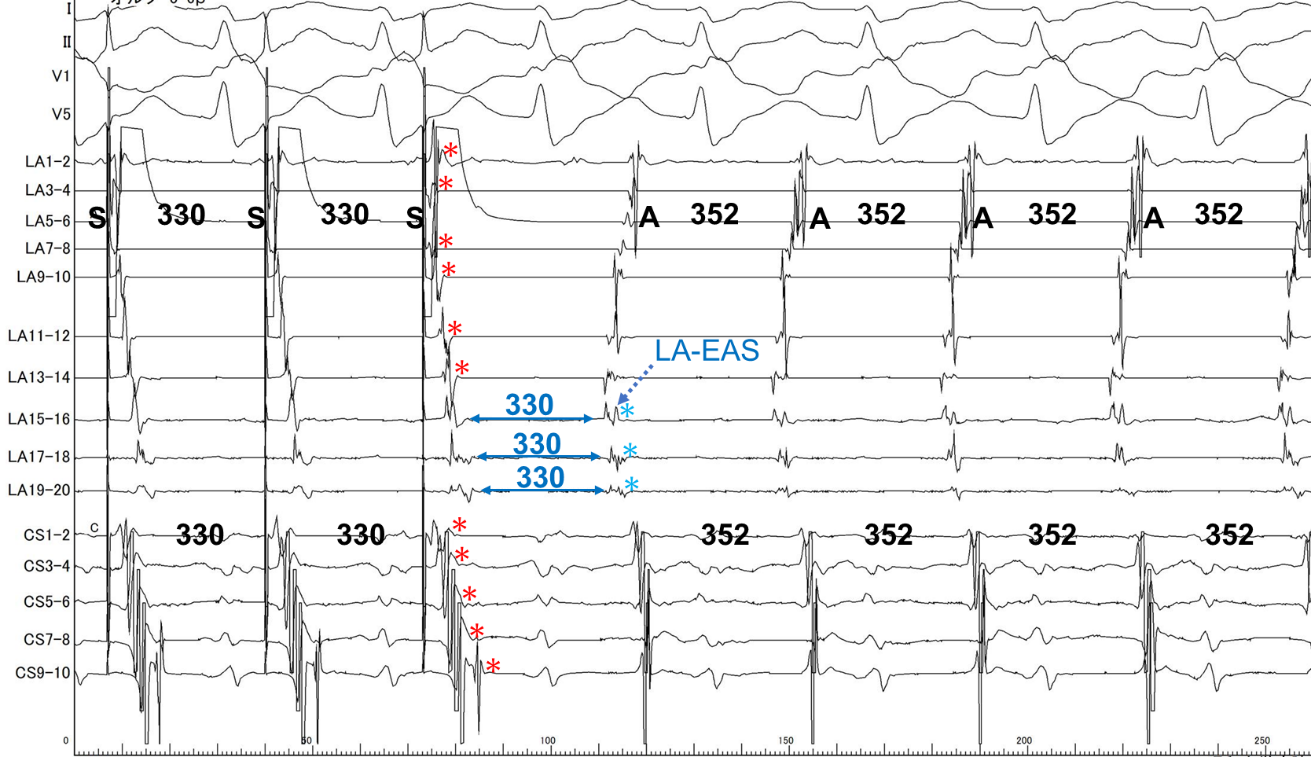


Supplemental Figure 3: Entrainment pacing from the base of LAA (LA 5-6). The LA-EAS was located at the LA15-16 electrodes. The cycle lengths of the tachycardia and pacing were 352 ms and 330 ms, respectively. The blue asterisks represent the last orthodromic captured electrograms, while the red asterisks represent the last antidromic captured electrograms. LA = left atrium; CS = coronary sinus; LA-EAS = left atrial earliest activation site; LAA = left atrial appendage.


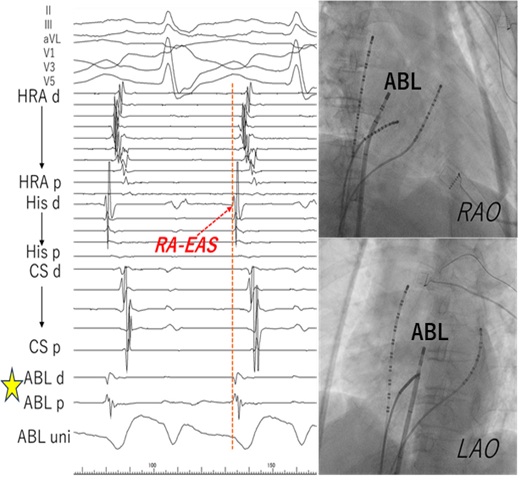


Supplemental Figure 4: Local electrograms (both bipolar and unipolar) and fluoroscopic images at the successful ablation site. No characteristic potentials, such as fragmented potentials, were observed from the endocardial side, supporting the possibility that the SCZ is located in the epicardial myocardium (interatrial septum). This site is 9.2 mm away from the LA-EAS, and the degree of early activation was equivalent to that of the RA-EAS. HRA = high right atrium; His = his bundle; CS = coronary sinus; ABL = ablation; d = distal; p = proximal; RA-EAS = right atrial earliest activation site; RAO = right anterior oblique view; LAO = left anterior oblique view.
