# Supplementary material for: Unusual left‐sided variant orientation of the slow conduction zone in adenosine‐sensitive atrial tachycardia
Source: J Arrhythm. 2025 Feb 7;41(1):e70015. doi: 10.1002/joa3.70015 (PMC11803878; doi:10.1002/joa3.70015)
Supplement: Supplementary file 2 — Video S1. [file JOA3-41-e70015-s001.zip › JOA370015-sup-0002-VideoS1/JOA370015-sup-0002-Video1.docx]

Video 1: The activation map of both atria during tachycardia are presented. The earliest activation site is located at the anterior septum of the left atrium.
